# Evaluating color stability and enamel surface roughness following resin infiltration treatment

**DOI:** 10.1002/cre2.834

**Published:** 2024-02-01

**Authors:** Mohammad Y. Sabti, Isra Y. Alfarhan, Aqdar A. Akbar, Muawia A. Qudeimat

**Affiliations:** ^1^ Department of General Dental Practice, College of Dentistry Kuwait University Kuwait Kuwait; ^2^ Department of Developmental and Preventive Sciences, College of Dentistry Kuwait University Kuwait Kuwait

**Keywords:** color, enamel, infiltrates, stain

## Abstract

**Objective:**

To assess the impact of resin infiltration treatment on enamel color stability and surface roughness after simulating daily exposure to coffee stains and regular toothbrushing with standard toothpaste.

**Materials and Methods:**

In this in vitro study, we utilized 47 caries‐free human enamel specimens, which were divided into two distinct groups. The teeth in the study group underwent artificial demineralization, Icon resin infiltration, and polishing, while the control group received only polishing. Following this, all teeth from both groups were regularly immersed in freshly brewed coffee for 15 min, twice daily, over a span of 30 days. After each staining session, the teeth underwent 10 heavy circular strokes using a soft toothbrush and standard toothpaste. Color changes were measured on Days 1 and 30 using a spectrophotometer. The L*, a*, b* color system defined by the International Commission on Illumination was used to assess the changes. Surface roughness was evaluated at baseline and after 30 days using a 3D surface Metrology Microscope.

**Results:**

After 30 days of immersion in coffee, both the study and control groups showed significant changes in color. However, differences were statistically significant between groups for “L” and “a” parameter but not for “b.” The overall color change in the study group was higher than the control group. There were no significant differences in surface roughness within or between the groups before and after staining and brushing.

**Conclusions:**

Resin infiltration leads to a significant increase in staining compared to untreated enamel. These staining properties should be taken into account when making clinical decisions regarding the treatment of white spot lesions.

## 1 INTRODUCTION

Dental caries is considered a worldwide high‐prevalence disease (Dorri et al., 2015; Meyer‐Lueckel et al., 2016; Paris, Schwendicke, Seddig, et al., 2013). Despite the caries being described as chronic and infectious, it is completely reversible and preventable in its early stages (Dorri et al., 2015). Early detection of caries along with nonoperative management approaches such as topical fluoride and fissure sealant applications are effective in reversing or stopping the progression of the disease (Abdullah & John, 2016; Ceci et al., 2017; Dorri et al., 2015; Leland et al., 2016). In recent years, resin infiltration, a minimally invasive approach for treating early caries lesions, has been introduced. It is considered a conservative approach that encourages the preservation of hard tissue and defers any surgical intervention of early carious lesions (Abdullah & John, 2016; Ceci et al., 2017; Gözetici et al., 2019; Turska‐Szybka et al., 2016). This technique involves tooth surface preparation via acid etching and placing a resin layer on the tooth surface to infiltrate the demineralized tissue (Abdullah & John, 2016; Dorri et al., 2015; Gözetici et al., 2019; Turska‐Szybka et al., 2016). Of the most important characteristics of the resin infiltration material are its low viscosity, high penetration coefficient, and a light‐cure command setting (Abdullah & John, 2016; Dorri et al., 2015; Gözetici et al., 2019; Meyer‐Lueckel et al., 2016; Paris, Schwendicke, Seddig, et al., 2013; Turska‐Szybka et al., 2016). This can facilitate deeper penetration of the material into the porosities of demineralized enamel, resulting in occlusion of these pores and thus arresting caries progression (Abdullah & John, 2016; Dorri et al., 2015; Gözetici et al., 2019; Meyer‐Lueckel et al., 2016; Paris, Schwendicke, Seddig, et al., 2013; Turska‐Szybka et al., 2016).

Clinical and laboratory studies have shown that resin infiltration has the ability to seal the carious lesion and prevent its progression (Altarabulsi et al., 2014; Gurdogan et al., 2017; Paris, Schwendicke, Seddig, et al., 2013). In a study that compared various approaches, researchers observed that resin infiltration outperformed other noninvasive methods in curbing the progression of carious lesions during an 18‐month timeframe (Gurdogan et al., 2017). A 4‐year retrospective study demonstrated no progression of all treated interproximal incipient lesions with resin infiltrates (Caglar et al., 2015). Altarabulsi et al. (2014) reported comparable results with only 4% caries progression in some lesions. Their conclusion was that resin infiltration represents an effective management choice that can reduce the progression of early interproximal caries (Altarabulsi et al., 2014). However, this technique is not without disadvantages. Staining has been reported to be one of the main disadvantages of the material, especially in the aesthetic zone (Caglar et al., 2015; Leland et al., 2016; Paris, Schwendicke, Keltsch, et al., 2013). As previously documented, beverages like wine and coffee have been shown to induce significant color changes in resin‐treated bovine enamel and dentin samples (Leland et al., 2016). A study reported that 19% of incipient lesions treated with resin infiltrates were stained (Caglar et al., 2015). The authors concluded that more research is needed to understand the performance and stability of the material under chemical, mechanical, and staining challenges (Leland et al., 2016). Therefore, the aims of this study were to test surface roughness and color stability of demineralized enamel surfaces treated with resin infiltration after simulating daily exposure to stains and mechanical manipulation. The null hypotheses were (1) demineralized enamel treated with resin infiltration has a similar surface roughness compared to healthy teeth after daily toothbrushing for 30 days and (2) demineralized enamel treated with resin infiltration stains similarly to untreated healthy enamel after daily exposure to coffee.

## 2 METHODS

**Figure 1:**
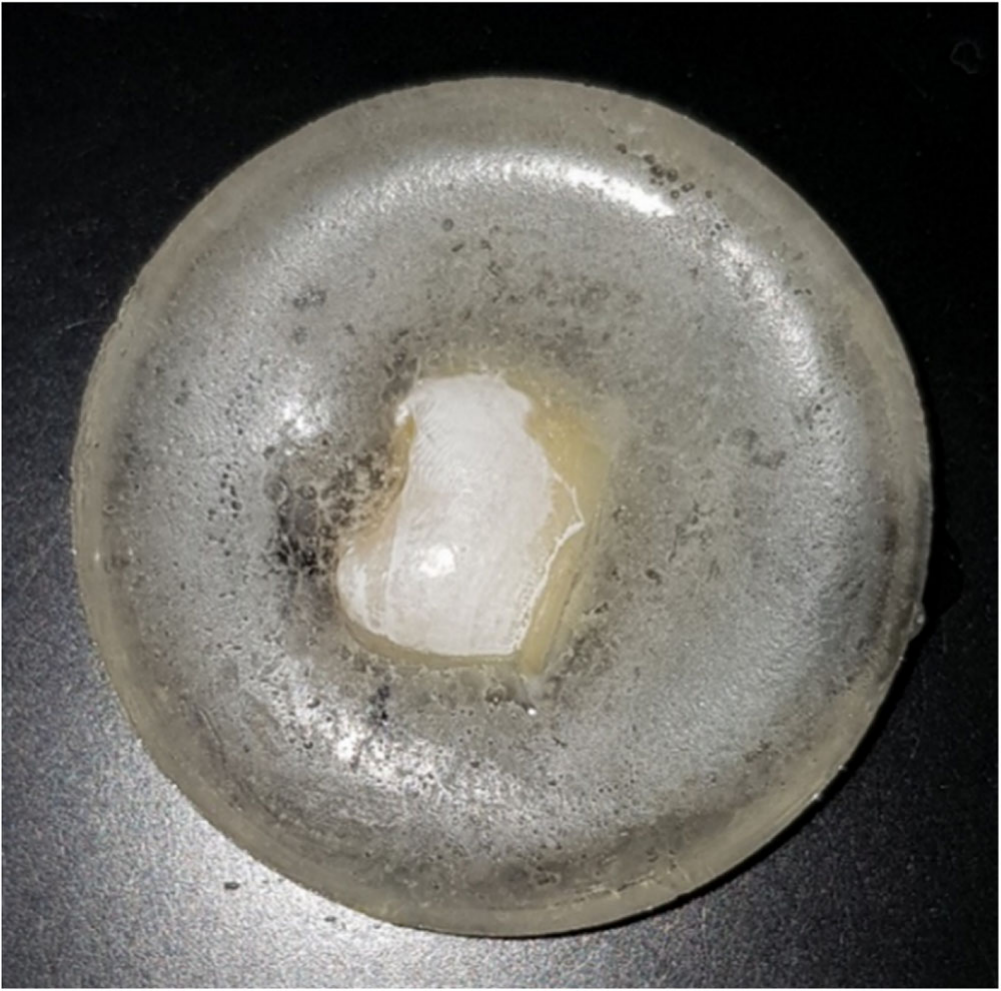
Enamel specimen mounted in cold‐curing acrylic.

The study has been approved by the Health Science Ethical Clearance Committee at Kuwait University (IRB: VDR/EC/3236), and all participants gave their informed consent to have their teeth collected for this study. The study was conducted in accordance with the Declaration of Helsinki. In total, 50 caries‐free extracted permanent human teeth were collected and stored in a 0.1% thymol solution for a period of no longer than 6 months. Inclusion criteria included freshly extracted, caries‐free, stain‐free, and unrestored permanent teeth with no signs of excessive tooth wear, developmental defects, or cracks. To clarify the presence or absence of enamel surface defects and cracks, the specimens were investigated using a stereomicroscope SteREO Discovery. V12 (Carl Zeiss MicroImaging GmbH, BioSciences). After applying the inclusion/exclusion criteria, 25 teeth were included in the study. The teeth were cleaned, the roots of each tooth were sectioned off, and the crowns were sectioned in half mesiodistally using a hard tissue microtome (Accutom‐100; Struers). From each tooth, a lingual and a buccal wide enamel over dentin sample were produced. These were mounted in cold‐curing orthodontic acrylic resin (Vertex Orthoplast, Holland Dental) that covered the dentin part of the specimen (Figure 1).

**Figure 2:**
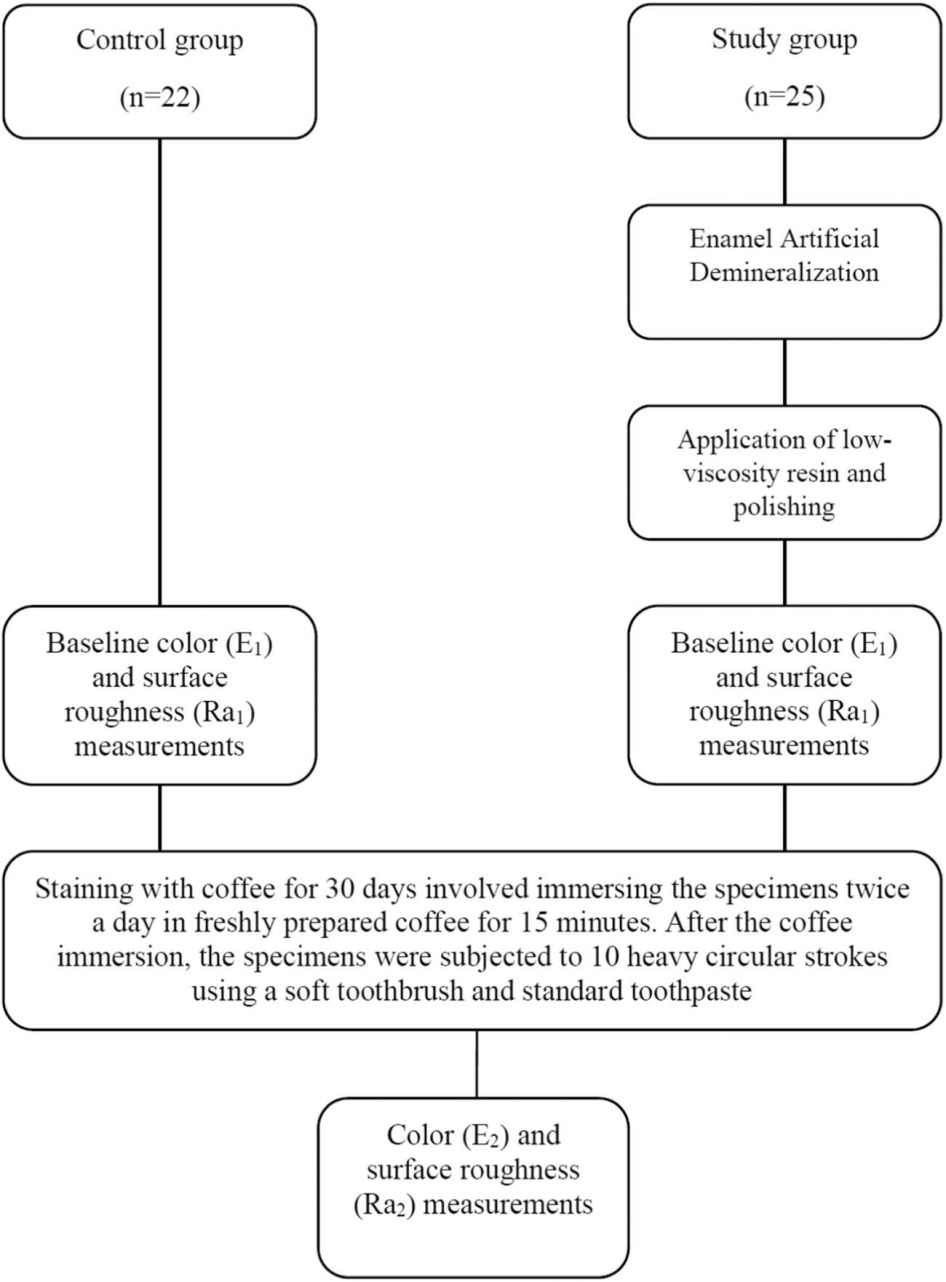
Overview of study design and procedure followed in this investigation.

The specimens were numbered and randomly divided into two groups: a control group (25 specimens) and a study group (25 specimens). Then, a second clarification for the presence of enamel cracks and surface contamination with epoxy was carried out using a stereomicroscope SteREO Discovery. V12. During the inspection of the samples, three specimens in the control group were found to be contaminated with mounting epoxy on the surface of the enamel being tested. Therefore, these samples were discarded, resulting in 22 samples in the control group. Figure 2 illustrates the experimental design of this study.

### 2.1 Enamel demineralization

**Figure 3:**
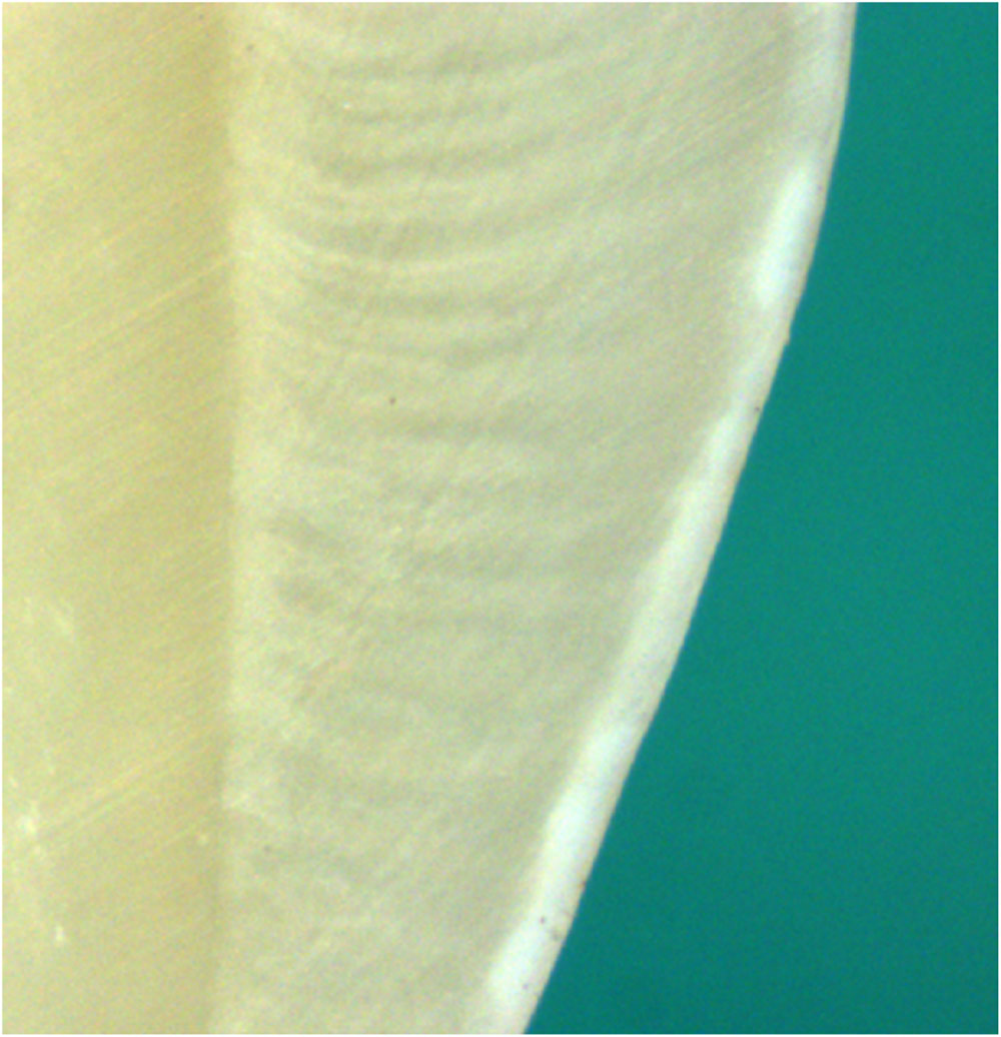
Enamel specimen showing artificial subsurface lesion.

To simulate white spot lesions, the teeth in the study group were subjected to artificial demineralization. A gel was prepared following the method described in a previous study (Al‐Kandari, 2017). This procedure entailed the addition of 0.1 M sodium hydroxide to a solution containing 0.1 M DL‐Lactic acid 87% (Sigma Aldrich) with the aim of achieving a pH level of 4.5. Subsequently, 6% w/v hydroxyethyl cellulose (Sigma Aldrich) was introduced into the solution and subjected to stirring for a duration of 1 h, resulting in a consistency similar to that of “wallpaper paste.” The resulting mixture was allowed to settle for a period of 24 h. Later, the demineralizing gel was transferred into universal tubes, within which the mounted teeth were immersed for further experimentation. To initiate the demineralization process, five samples underwent immersion in the solution for a duration of 9 days. Following this period, the samples underwent sectioning, and the subsurface lesions were quantified using a microscope. The results showed an average depth of 50 microns for the samples (Figure 3). These samples were excluded from the study. The teeth in the study group were then immersed in the demineralizing gel and stored at room temperature for 9 days to produce an artificial enamel subsurface lesion. After demineralization, the enamel samples from the study group were removed from the acid gel, washed, and stored in distilled water at 8°C.

### 2.2 Icon infiltration

The manufacturer's instructions were followed when performing resin infiltration. For the control samples (*n* = 22), no demineralization was done, and no Icon was applied. For the experimental group (*n* = 25), 15% hydrochloric acid gel (Icon‐Etch; DMG) was applied on the artificially demineralized enamel surface for 2 min, then rinsed with water and dried with air for 30 s. This was followed by the application of Icon‐Dry (ethanol) for 30 s, and an additional air drying. This procedure was repeated twice. Then, Icon‐Infiltrant (low‐viscosity resin) was applied twice. The first layer was applied for 3 min and light‐cured with Optilux 501 (Kerr/Demetron) for 40 s. The second layer was applied for 1 min and light‐cured for 40 s. Samples were polished with Diacomp, a two‐step polishing system (EVE Ernst Vetter GmbH), using a slow‐speed handpiece at 3000 RPM.

### 2.3 Teeth staining

Specimens were placed in 4 mL of freshly made and hot Nescafé coffee infusion (powder: water ratio, 25 g: 250 mL) for 15 min to simulate extrinsic dietary staining. Then the samples were removed and brushed by the same investigator (M. S) with a soft toothbrush (Oral‐B, Procter & Gamble) with standard toothpaste (Sensodyne ProNamel Daily Protection, Haleon UK Trading) for 10 heavy strokes in a circular motion. Following this, the specimens were placed in distilled water at a room temperature of 24°C. This process was repeated twice a day, with a minimum of 6 h between the first and second procedure, for 30 days.

### 2.4 Color assessment

To test the color changes, a spectrophotometer (Vita. Easyshade V, Vident) was used (Lath et al., 2007). For each specimen, the baseline value (E_1_) before staining in the control group and 48 h after Icon applications in the study group (to account for rehydration) were recorded. The final color assessment for both groups (E_2_) was done after 30 days of staining with coffee. All color measurements were carried out under standardized ambient conditions. A daylight bulb was used to standardize the room lighting, and a white background was used for all specimens during the readings. The tip of the spectrophotometer was always held in a vertical position while being supported on the surface of the specimens. The quantification of color changes in the results was carried out using three coordinate values (L*, a*, b*), in accordance with the standards set by the International Commission on Illumination. Specifically, L* indicates the degree of color lightness within a specimen, with a range from 0 to 100, where 0 represents black, and 100 represents white. On the other hand, a* and b* signify chromaticity without specific numerical limits. A negative value of a* corresponds to green, a positive value corresponds to red, while a negative value of b* corresponds to blue, and a positive value corresponds to yellow. This method helps locate the color of an object in a 3‐dimensional color space. The changes in the values of L* (ΔL_2__−__1_), a* (Δa_2__−1_), and b* (Δb_2__−1_) were assessed and the final change in color (ΔE_2__−1_) was determined according to the following formula:

ΔE_2__−1_ = [(L_2_−L_1_)^2^ + (*a*_2_−*a*_1_)^2^ + (*b*_2_−*b*_1_)^2^]^0.5^. The color of each specimen was read three times, and the values were averaged.

### 2.5 Surface roughness measurements

Surface roughness was characterized by amplitude/height (Sa, Sq, Sz, and Sv) and functional parameters (Smc, Smr, and Sxp) (Vieira et al., 2023). To facilitate the standardization of surface roughness testing, a horizontal groove close to the flattest part of the enamel was created on the acrylic block at baseline. This ensured that the readings at baseline and after 30 days were made in the same location on each specimen. Surface roughness testing was performed using a 3D Optical Surface Metrology Microscope DCM8 (Leica Microsystems). Samples were scanned with a Leica EPI 10× magnification lens with a field of view of 1.5 × 1.5 mm, and a scan speed of 1×. The images were viewed on Leica SCAN (Leica) software to analyze the images. Each sample underwent scanning at three designated points, and the resulting values were averaged to calculate the height and functional surface roughness parameters. In the case of the control group, surface roughness was initially measured before the commencement of the experiment (Sa_1_, Sq_1_, Sz_1_, Sv_1_, Smc_1_, Smr_1_, and Sxp_1_) and subsequently after 30 days of staining and brushing (Sa_2_, Sq_2_, Sz_2_, Sv_2_, Smc_2_, Smr_2_, and Sxp_2_). Similarly, for the study group, the surface roughness was measured after Icon application and again after 30 days of staining and brushing.

### 2.6 Statistical analysis

The data were input and organized in a computer using Statistical Package for Social Science (SPSS) version 23.0 for Windows, Chicago, IL, USA. Data analysis encompassed descriptive statistics, paired *t* tests, chi‐square tests, and analysis of variance. Statistical significance was defined as a probability value of ≤.05.

## 3 RESULTS

The differences in color parameters (L, a, and b) between the two groups were not statistically significant at baseline (*p* > .05). However, after immersing in coffee for 30 days, significant color changes were observed. Both the study and control groups demonstrated statistically significant changes in color lightness (ΔL) and color chromaticity (Δa and Δb) compared to their baseline measurements after 30 days of coffee staining (Table 1).

**Table 1:** The mean and SD changes in color lightness (ΔL) and color chromaticity (Δa and Δb) before and after coffee staining.

Group	Color parameter	Baseline mean (SD)	30 days after staining mean (SD)	Mean difference (SD)	*df*	95% CI	*p* Value
Control	L	79.43 (4.62)	64.66 (4.39)	14.78 (4.59)	21	12.74–16.81	<.0001
	a	−0.52 (1.48)	2.36 (1.15)	−2.88 (1.83)	21	−3.69 to −2.07	<.0001
	b	25.42 (6.60)	30.24 (4.24)	−4.83 (1.05)	21	−7.01 to −2.65	<.0001
Study	L	82.97 (5.01)	56.61 (7.24)	26.36 (8.47)	24	22.86–29.86	<.0001
	a	−1.07 (0.77)	5.21 (2.24)	−6.28 (2.24)	24	−7.20 to −5.35	<.0001
	b	23.16 (4.11)	34.17 (4.11)	−11.00 (4.81)	24	−12.99 to −9.02	<.0001

Abbreviations: *df*, degrees of freedom; SD, standard deviation.

The most notable change observed in both experimental groups was a decrease in color lightness (L* value) after 30 days of coffee exposure. The mean difference in the L* value was 14.78 ± 4.59 for the control group and 26.36 ± 7.24 for the study group. Additionally, both groups exhibited a shift in the a* value from a negative value baseline to a positive value after 30 days (−0.52 ± 1.48 to 2.36 ± 1.15 in the control group and −1.07 ± 0.77 to 5.21 ± 2.24 in the study group), indicating a change in color chromaticity from green to red. Moreover, the b* value consistently remained positive, suggesting an increase in the chromaticity of yellow color after 30 days of coffee staining.

Regarding the intergroup comparison, the differences in the “L” and “a” parameters were statistically significant (*p* < .05), whereas the “b” parameter did not demonstrate a statistically significant difference between the two groups. The overall color change (ΔE_2__−__1_) after coffee staining in the control group (ΔE_2__−1_ = 16.38, SD = 5.41) was lower than that in the study group (ΔE_2__−1_ = 27.18, SD = 7.56), and this difference was statistically significant (F = 30.91, *p* < .0001).

The results of the surface roughness test for both groups are shown in Table 2. There were no significant differences within or between the groups in the average surface roughness measured before staining and brushing and after 30 days of staining and brushing in either group (*p* > .05).

**Table 2:** Paired *t* test for the mean difference of surface roughness height parameters within each group, and a two‐way analysis of variance between the two groups after (2) and before (1) staining and brushing over 30 days.

Surface roughness test	Group control	Study	F	*p* Value
**Sa1**	2.23 (1.53)	2.00 (0.68)	0.46	.50
**Sa2**	1.96 (0.44)	2.10 (0.46)	1.16	.29
Mean difference (SD)	−0.27 (1.52)	0.10 (0.93)		
95% CI	−0.94–0.40	‐0.28–0.48		
*p* Value	0.41	0.59		
**Sq1**	2.68 (1.13)	2.68 (0.92)	0.00	.99
**Sq2**	2.67 (0.66)	2.79 (0.62)	0.40	.53
Mean difference (SD)	−0.003 (0.94)	0.12 (1.14)		
95% CI	−0.42–0.42	−0.35–0.59		
*p* Value	0.99	0.61		
**Sz1**	24.50 (16.93)	23.10 (10.34)	0.12	.73
**Sz2**	23.90 (11.71)	28.28 (11.47)	0.16	.69
Mean difference (SD)	−0.59 (15.83)	2.18 (14.49)		
95% CI	−7.61–6.42	−3.80–8.16		
*p* Value	0.86	0.46		
**Sv1**	26.10 (23.45)	17.14 (15.16)	2.48	.12
**Sv2**	23.90 (11.71)	16.29 (14.50)	3.85	.06
Mean difference (SD)	−2.20 (21.43)	−0.86 (18.73)		
95% CI	−11.7–7.30	−8.59–6.87		
*p* Value	0.64	0.82		
**Smc1**	3.07 (1.38)	3.14 (1.28)	0.03	.86
**Smc2**	2.83 (0.82)	3.11 (1.51)	0.88	.35
Mean difference (SD)	−0.24 (1.18)	−0.03 (1.77)		
95% CI	−0.76–0.29	−076–0.70		
*p* Value	0.36	0.94		
**Smr%1**	0.03 (0.08)	0.34 (1.44)	1.02	.32
**Smr%2**	0.04 (0.17)	0.01 (0.008)	1.07	.31
Mean difference (SD)	0.01 (0.12)	−0.34 (1.44)		
95% CI	−0.04–0.06	−0.93–0.26		
*p* Value	0.70	0.25		
**Sxp1**	5.08 (1.52)	4.82 (1.63)	0.32	.57
**Sxp2**	5.54 (1.15)	5.07 (1.31)	1.72	.20
Mean difference (SD)	0.46 (1.93)	0.25 (2.09)		
95% CI	−0.40–1.31	−0.61–1.11		
*p* Value	0.28	0.56		

Abbreviations: CI, confidence interval; SD, standard deviation.

## 4 DISCUSSION

The color of resin restorations, especially in direct restorative materials, significantly influences patient satisfaction and restoration aesthetics, ultimately affecting longevity (Dietschi et al., 1994). Research highlights that resins with low resin content, a high filler‐resin ratio, and reduced particle size exhibit enhanced color stability (Dietschi et al., 1994). Conversely, nonfilled low‐viscosity resins, as seen in resin infiltration, may face aesthetic challenges over time, particularly with exposure to staining agents like coffee (Caglar et al., 2015; Leland et al., 2016; Topcu et al., 2009). Our study assessed the staining impact of coffee, a widely consumed drink known to discolor tooth structure and resin materials, on resin infiltrates (Park et al., 2010; Topcu et al., 2009). To further simulate real‐life conditions, we incorporated manual toothbrushing twice a day over a 30‐day period.

Several subjective and objective methods are available to evaluate tooth color changes, such as visual comparisons with tooth color shade guides, image analysis, colorimeters, or spectrophotometers (Chu et al., 2010; Joiner, 2006). An objective method (spectrophotometer) was used in the present study to quantitatively analyze the data. The results of the color readings were quantified in terms of three coordinate values (L*, a*, b*). This helped in locating the color of an object in a 3‐dimensional color space, with the “L*” (lightness of the object) being the most relevant for comparison under experimental conditions (Dietschi et al., 2010). L* value of zero indicates a deep black color and 100 indicates a perfect reflecting diffuser (Joiner, 2006). Additionally, the overall change in color (ΔE) was calculated, which gives an indication of the color changes within and between the two groups at different moments, providing an overall color change (Caglar et al., 2015).

Like other similar studies, immersing the enamel specimens in coffee for 30 days resulted in a significant color change in the two study groups, with the L* value being the most affected parameter (Araújo et al., 2015; Borges et al., 2014; Neres et al., 2017). The lightness significantly dropped by 14.8 in the control group and by 26.4 in the resin infiltrate group, indicating the incorporation of the brown dye from the coffee into the teeth and resin surfaces, which also resulted in a significant increase in both a* and b* values. A previous study found that the L* values of sound enamel were significantly higher than those of enamel infiltrated with Icon, indicating that the infiltrated enamel is highly susceptible to dye penetration (Araújo et al., 2015).

In this study, the overall increase in color was statistically significantly higher in the study group than in the control group, suggesting that resin‐infiltrated teeth stain significantly more than natural dentition over time. The same results were previously observed, where compared to sound enamel, white spot resin‐infiltrated lesions demonstrated significantly greater discoloration after being subjected to pH cycling, long‐term thermocycling, staining, and toothbrushing (Neres et al., 2017). Similar findings were also observed when bovine teeth were immersed in wine, coffee, and water (Dietschi et al., 2010). The authors concluded that the changes in color were significantly higher in the wine and coffee groups when compared with immersing the specimens in water. In the previous study, the highest staining values were detected in the resin‐infiltrated group. These findings can be attributed to the composition of Icon (Borges et al., 2014). Although the exact components of Icon are not well described by the manufacturer, the material exhibits high water sorption which makes absorption of dyes from food and beverages a disadvantage for the material (Araújo et al., 2015; Borges et al., 2014).

Under the current study conditions, the second hypothesis was accepted, as there was no significant difference in surface roughness before and after staining and brushing within or between the two groups. These results may suggest that the differences in color changes in the samples of this study were due to the chemical properties of the material rather than surface roughness. Our findings are in agreement with a previous investigation, where the author found that, after subjecting enamel treated with resin infiltrate to 10,000 strokes of toothbrushing over a total of 45 min, no difference was found between the surface roughness of infiltrated enamel and infiltrated enamel subjected to brushing (Neres et al., 2017). On the other hand, the results of our study are in disagreement with a study where the investigators reported that, resin infiltration reduced the surface roughness after exposure to multiple challenges including coffee staining, but the values of sound enamel at baseline were not reached (Liu et al., 2022). Another study measured surface roughness in incisors treated with resin infiltration and compared it to sound enamel (Gurdogan et al., 2017). It was concluded that Icon infiltration created a significantly rougher surface when compared to sound enamel (Gurdogan et al., 2017). The investigators suggested that this was due to the presence of demineralized prismatic areas that were not filled by the infiltrating resin due to polymerization shrinkage or due to the interference of ethanol in polymerization (Neres et al., 2017). Similarly, when measuring the surface roughness of human third molar teeth, it was observed that the surface roughness of sound enamel was less than that of resin‐infiltrated lesions (Zhao & Ren, 2016). The variations in surface roughness seen across different studies can be attributed to differences in the protocols followed. While our study aimed to replicate real‐life situations of daily coffee staining and manual toothbrushing over 30 days, other studies utilized mechanical brushing machines to simulate years of service (Neres et al., 2017), pH cycling (Gurdogan et al., 2017; Liu et al., 2022), thermocycling (Liu et al., 2022; Zhao & Ren, 2016), and others used bovine enamel samples (Gurdogan et al., 2017; Liu et al., 2022; Neres et al., 2017) creating variations in the obtained results.

This study is not without limitations, as the artificial demineralization method and staining protocol may not fully replicate natural oral processes, potentially impacting results influenced by various oral environment challenges and oral healthcare products (Celik & Iscan Yapar, 2021; Forcin et al., 2021; Liu et al., 2022; Neres et al., 2017; Thimmaiah et al., 2019). Although a previous study found no significant difference in surface roughness after subjecting infiltrated enamel to brushing and artificial aging challenges (Neres et al., 2017), the method's generalizability remains a concern. Additionally, the study's focus on a single drink stain limits its representativeness of diverse staining sources (Borges et al., 2014; Leland et al., 2016). Moreover, exclusive reliance on a spectrophotometer for color assessment, without human visual assessments or established perceptibility (PT) and acceptability (AT) thresholds, presents a limitation. Notably, the controversy surrounding PT and AT thresholds is acknowledged, with studies reporting diverse values for PT (PT/∆E range of 0.4–3.7) and for AT (AT/∆E range of 2–4) (Khashayar et al., 2014). In addition, future research should explore different brushing cycles to understand their impact on surface roughness and resin infiltrate discoloration. Despite these limitations, this study contributes valuable insights into resin infiltrates discoloration under specific conditions.

## 5 CLINICAL RELEVANCE

### 5.1 Scientific rationale for the study

Staining has been reported as one of the main drawbacks of resin infiltration in resin‐treated bovine teeth. This study aimed to examine the color stability and surface roughness of human enamel treated with resin infiltration after exposure to coffee stains and toothbrushing with standard toothpaste, with the goal of evaluating the staining properties and their impact on clinical decision‐making.

### 5.2 Principal findings

Significant statistical differences between the groups were observed in terms of color parameters, indicating that resin infiltration led to more staining compared to untreated enamel.

### 5.3 Practical implications

When treating white spot lesions, the staining properties of resin infiltrants should be taken into account during clinical decision‐making. Resin infiltration was found to cause more staining than untreated enamel, emphasizing the importance of improving the properties of the material or considering alternative treatment options. Additionally, the study found that resin infiltration did not negatively affect the enamel surface texture, indicating its suitability for clinical use without compromising surface roughness.

## AUTHOR CONTRIBUTIONS

All authors contributed to the present study. Mohammad Y. Sabti, Aqdar A. Akbar, and Muawia A. Qudeimat contributed to the conception and design. Mohammad Y. Sabti, Aqdar A. Akbar, Isra Y. Alfarhan, and Muawia A. Qudeimat contributed to material preparation and data analysis and interpretation. All authors gave final approval and agreed to be accountable for all aspects of the work.

## CONFLICT OF INTEREST STATEMENT

The authors declare no conflict of interest.

## Data Availability

The data that support the findings of this study are available from the author (M. Y. S.) upon reasonable request.
